# Electroacupuncture Inhibits Inflammation Reaction by Upregulating Vasoactive Intestinal Peptide in Rats with Adjuvant-Induced Arthritis

**DOI:** 10.1155/2011/290489

**Published:** 2010-09-14

**Authors:** Tian-Feng He, Wen-Jia Yang, Shu-hui Zhang, Chun-Yan Zhang, Lian-Bo Li, Yun-Fei Chen

**Affiliations:** ^1^Institute of Acupuncture-Moxibustion and Meridian, Shanghai University of Traditional Chinese Medicine, Shanghai 200030, China; ^2^Laboratory Center of Medicine, Yueyang Hospital of Integrated Traditional Chinese and Western Medicine, Shanghai University of Traditional Chinese Medicine, Shanghai 200437, China

## Abstract

Acupuncture is emerging as an alternative therapy for rheumatoid arthritis (RA). However, the molecular mechanism underlying this beneficial effect of acupuncture has not been fully understood. Here, we demonstrated that electroacupuncture at acupoints Zusanli (ST36), Xuanzhong (GB39); and Shenshu (BL23) markedly decreased the paw swelling and the histologic scores of inflammation in the synovial tissue, and reduced the body weight loss in an adjuvant-induced arthritis rat model. However, the electrical stimulation at nonacupoint did not produce any beneficial effects against the experimental arthritis. Most interestingly, the electroacupuncture treatment resulted in an enhanced immunostaining for vasoactive intestinal peptide (VIP), a potent anti-inflammatory neuropeptide, in the synovial tissue. Moreover, the VIP-immunostaining intensity was significantly negatively correlated with the scores of inflammation in the synovial tissue (*r* = −0.483, *P* = .0026). In conclusion, these findings suggest that electroacupuncture may offer therapeutic benefits for the treatment of RA, at least partially through the induction of VIP expression.

## 1. Introduction

In China, acupuncture has been used to treat a variety of health problems for more than 5000 years. Nowadays, this traditional Chinese medical technique has become very popular worldwide as a complementary medicine [1]. Some of the common applications of acupuncture include heart disease [2], smoking cessation [3], and the treatment of inflammatory diseases such as rheumatoid arthritis (RA) [4–7], autonomic dysfunction [8–10], neurological diseases [11], neuropathic pains [12–14], drug addiction [15, 16], and psychological disorders [17, 18]. Although neuroendocrine-immune, gene expression; and proteomic analysis have been employed and provided some molecular interpretations in many experiments about electroacupuncture (EA), the molecular mechanism of acupuncture is still not clear [19].

RA is a systemic inflammatory disorder that mainly affects the diarthrodial joint. Although acupuncture has been clinically used to treat the patients with RA, the mechanism underlying the beneficial effects of acupuncture is still unclear. Some researchers have suggested that the action of acupuncture may be associated with the improvement of tissue function through vasodilatation in the skin due to axon reflexes [20]. Neuroendocrine-immune interactions have been proposed to contribute to the pathogenesis of RA. Vasoactive intestinal peptide (VIP) has been found to act as a potent anti-inflammatory factor in experimental arthritis through regulating the production of both anti- and proinflammatory mediators and promoting Th2-type responses [21]. Additionally, VIP can negatively regulate the Toll-like receptor 4-signaling in rheumatoid arthritis synovial fibroblasts [22]. Many studies have documented that the VIP levels can be modulated by acupuncture. Acupuncture at Zusanli (ST36) has been reported to cause an elevation of VIP levels in plasma, gastric mucosal and, bulb tissues in rats [23]. In contrast, EA at Tianshu (ST-25) in combination with Tegaserod administration into the stomach can inhibit the secretion of VIP in rats with irritable bowel syndrome [8]. Given the modulation of VIP by acupuncture and the importance of VIP in experimental arthritis, we hypothesized that the action of acupuncture on RA may be attributable to the elevation of VIP expression and the subsequent suppression of the arthritis. To test this hypothesis, we established a Freund's complete adjuvant- (FCA-) induced arthritis model in rats and evaluated the effects of EA at Zusanli (ST36), Xuanzhong (GB39) and Shenshu (BL23) on the arthritis and the expression of VIP. In addition, the relationship between the inflammation scores and VIP levels was analyzed. The above acupoints were chosen in this study because they are commonly used in the treatment of arthritis through acupuncture, and they have the effects of anti-inflammatory, reduce pain, and modulate neuroendocrine-immune system [20, 24–27].

## 2. Materials and Methods

### 2.1. Animals

Wistar rats (male, 5-6 weeks old) were purchased from Shanghai SLAC laboratory animal co., Ltd., Shanghai, China. Following adaptation for 1 week, rats were randomly divided into 5 groups (*n* = 15 for each group): the saline control group (injection of saline), the FCA group (injection of FCA to induce arthritis), the FCA-EA group (injection of FCA followed by EA stimulation at ST36, GB39, and BL23), the saline-EA group (injection of saline followed by EA stimulation at ST36, GB39, and BL23), and the FCA-EA (nonacupoint) group (injection of FCA followed by EA treatment at a nonacupoint). All experimental manipulations were undertaken in accordance with the National Institutes of Health Guide for the Care and Use of Laboratory Animals, and with the approval of the Scientific Investigation Board of the Shanghai University of Traditional Chinese Medicine, Shanghai, China.

### 2.2. Induction of Arthritis by FCA

Experimental arthritis was induced as described previously [28, 29]. Briefly, rats were anesthetized with 4% halothane in oxygen, and the right hindlimb footpad received an injection of 0.1 mL FCA (Sigma, St. Louis, MO, USA), which contains 0.1 mg heat-killed and dried Mycobacterium tuberculosis (strain H37Ra, ATCC 25177), 85 *μ*L paraffin oil and 15 *μ*L mannide monooleate. Rats in the control groups were injected with equal volume of saline instead of FCA. The inoculation day was designated as day 0.

### 2.3. EA Stimulation

**Figure 1:**
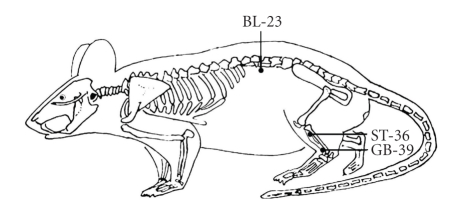
Schematic diagram indicates the three selected acupoints, Xuanzhong (GB39), Shenshu (BL23) and Zusanli (ST36), which correspond to equivalent acupoints in humans.

Three days following the FCA administration, EA stimulation was performed as described previously [27]. Briefly, small cages with five holes for four limbs and a tail were built. During the EA treatment, animals were maintained within the cage with right hind limb taken out and fastened to the wall of the cage with tape. Sterilized disposable stainless steel acupuncture needles (0.30 × 25 mm, Suzhou Kangnian Medical Devices Co., Ltd., Suzhou, China) were inserted perpendicularly as deep as 2-3 mm at Xuanzhong (GB39), 6 mm at Shenshu (BL23), and 7 mm at Zusanli (ST36). The depth of needle insertion into each acupoint was arbitrarily determined based on several previous studies [24, 30, 31]. The locations of the acupoints were illustrated in Figure 1; that is, GB39 is located at the distal 4/5 point on the line connecting the lateral side of the knee and the lateral malleolus of the tibiofibula, BL23 at the depression lateral to the lower border of spinous process of the second lumbar vertebra, and ST36 at the proximal 1/5 site of craniolateral surface of the leg distal to the head of the tibia in a depression between the muscles of the cranial tibia and the long digital extensor. The needles inserted into the acupoints were connected to an EA stimulator (Model LH 202H HANS, Beijing Huawei LTD, Beijing, China), and a constant electrical stimulus (2 Hz, 0.2 ms pulse width) was applied for 15 minutes. The intensity (6-7 mA) was adjusted until local muscle contractions were seen. The EA treatment was given once every other day for 15 days. To control for the possible effects of EA, some animals received an electrical stimulation at a nonacupoint (the gluteal muscle), following the same procedures as above. Additionally, the animals without receiving EA stimulation were restricted in cages in the same manner with the EA-stimulated animals.

### 2.4. Measurements of the Body Weight and the Paw Volume

The body weight and the paw volume for each rat were examined every 3 days for 18 days after adjuvant injection. The paw volume was blindly measured using a water displacement plethysmometer (YLS-7A, Yiyan Sci Ltd., Jinan, China). The mean values were calculated and plotted at each time point.

### 2.5. Hematoxylin and Eosin (H&E) Staining and Inflammation Scoring

The ankle joint was removed from each rat, washed in PBS, and fixed with 10% formalin in PBS. Paraffin-embedded sections (4 *μ*m) were deparaffinized in xylene followed by rehydration through an ethanol gradient to distilled water. H&E staining was performed on all samples for pathological examination. The stained sections were scored by 2 investigators who were blinded to the treatments, according to the following scale. 0, no inflammation; 1: mild inflammatory infiltrate in skin and overlying tissues; 2: dense inflammatory infiltrate but no synovitis or arthritis; 3: synovitis; 4: hyperplastic synovium, inflammatory infiltrate in the joint; 5. arthritis with destruction of articular tissues, pannus formation.

### 2.6. VIP Radioimmunoassay

The concentration of VIP in the serum of rats was determined by radioimmunoassay according to the manufacturer's protocol. [^125^I]-VIP for the preparation of the labeled VIP and synthetic VIP as standard were purchased from the Institute for Neuroscience, Second Military Medical University (Shanghai, China). All serum samples were assayed in duplicate and the mean values were calculated. Individual controls for nonspecific binding in the absence of antibody were also included. The radioimmunoassay results were expressed as nanograms of immunoreactive peptide per milliliter of serum (pg/mL).

### 2.7. RNA Isolation and Real-Time Quantitative RT-PCR (qPCR)

Total RNA was extracted from synovial tissues with an RNeasy mini kit (Qiagen, Germany) according to the manufacturer's protocol, and cDNA was subsequently synthesized from total RNA using the cDNA Synthesis kit (Roche, Mannheim, Germany). The qPCRs for the *VIP* or for the internal control glyceraldehyde-3-phosphate dehydrogenase (*GAPDH*) were performed using SYBR-green detection of PCR products in an ABI-7500 Real-time Cycler (ABI, Foster City, CA, USA). The cycling conditions were as follows: initial denaturation at 95°C for 10 minutes, then 40 cycles of denaturation at 95°C for 5 seconds, annealing at 60°C for 20 seconds, and elongation at 72°C for 15 seconds. The sequences of the primers for qPCR were as follows: sense 5′-TCA TTG GCA AAC GAA TCA GT-3′ and antisense 5′-ATT TGC TTT CTA AGG CGG GT-3′ for *VIP*, and sense 5′-GGC ATC CTG ACC CTG AAG TA-3′ and antisense 5′-GGG GTG TTG AAG GTC TCA AA-3′ for *GAPDH*. All qPCRs were performed in triplicate. The relative amount of *VIP*, normalized to the internal control *GAPDH* and relative to a calibrator, was calculated according to a previously described method [32].

### 2.8. Immunohistochemistry

Formalin-fixed, paraffin-embedded sections were deparaffinized and rehydrated in graded alcohol, and antigen retrieval was achieved using microwave heating. Sections were incubated with the primary antibody against VIP (diluted 1 : 500) overnight at 4°C, followed by the incubation with EnVision Plus System (Dako) as previously described [33]. Negative controls were included by omitting the primary antibody, and positive controls were added by including a known positive tissue. The stained sections were independently assessed by two pathologists without prior knowledge of the experimental protocols. The immunostaining for VIP was considered positive when the cytoplasm was stained brown. The levels of immunoreactive VIP protein were quantified morphometrically using Image Pro-Plus 6.0 software (Media Cybernetics Inc., USA) on an Olympus BX51 microscope (Japan). Five random fields on each slide at 400x magnification were analyzed, and the mean optical density was calculated.

### 2.9. Statistics Analysis

All statistical calculations were carried out using SPSS 13.0 software (SPSS, Chicago, IL, USA). The data were presented as mean ± SD. Statistical analysis was evaluated by one-way ANOVA and post hoc tests. Spearman bivariate correlation was used to determine the relation between the VIP protein expression and inflammation scores in the synovial tissue. A level of *P* < .05 was considered statistically significant.

## 3. Results

### 3.1. Alleviation of the FCA-Induced Body Weight Loss and Paw Swelling

**Figure 2:**
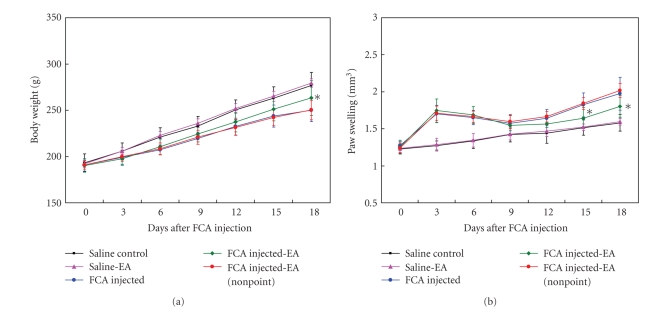
Effect of EA on the body weight and the paw swelling in adjuvant-induced arthritis Rats. (a) The body weight. *P < .05, compared with FCA-injected group. (b) The paw swelling. *P < .05, compared with FCA-injected group. Data are means ± SD (*n* = 15).

The average body weight was significantly decreased in the animals with adjuvant-induced arthritis as compared with that in the saline control group. The EA at acupoints substantially alleviated the body weight loss caused by the FCA injection (*P* < .05 versus the FCA group; Figure 2(a)). However, the electrical stimulation at nonacupoint had little influence on the FCA-elicited body weight reduction. Accompanying with the body weight loss, a pronounced induction of paw swelling was found following the FCA injection (Figure 2(b)). Interestingly, the FCA-induced paw swelling was significantly lowered by the EA at acupoints (*P* < .05 versus the FCA group), but not by the EA at nonacupoint. Additionally, the EA stimulation at the studied acupoints did not elicit any significant effects in saline-treated animals.

### 3.2. The Attenuation of FCA-Induced Inflammation

**Figure 3:**
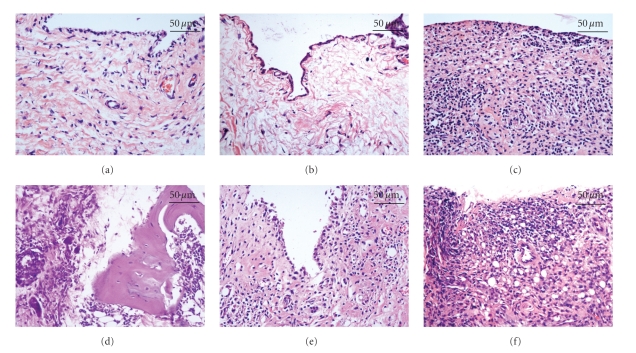
Histological study of the inflammation of the synovial tissue. A very mild inflammation in the ankle joint from the animals in the saline (a) and saline-EA (b) groups. Massive accumulation of inflammatory cells in the swollen joint (c) and an apparent periosteal edema (d) are seen in the FCA-injected group. (e) Less inflammation and hyperplastic synovial cells are presence in the ankle joint of the animals in the FCA-EA group. (f) A severe inflammation of the ankle joint is detected in the FCA-EA (nonacupoint) group. H&E staining; original magnification, 400x.

**Figure 4:**
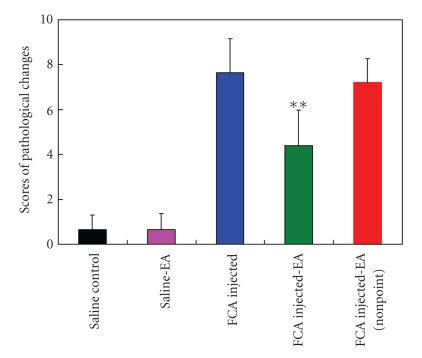
Histological scores of inflammation in the synovial tissue from the indicated groups. **P < .01, compared with the FCA group. Data are means ± SD (*n* = 15).

Histological analysis of sections of the ankle joint revealed little or no sign of inflammation in the saline (Figure 3(a)) and saline-EA (Figure 3(b)) groups. In contrast, the FCA-injected animals displayed massive accumulation of inflammatory cells in the swollen joint (Figure 3(c)) and an apparent periosteal edema (Figure 3(d)). The FCA-induced joint inflammation was considerably inhibited by the EA stimulation (Figure 3(e)). However, the electrical stimulation at nonacupoint did not offer any beneficial effects on the control of inflammation (Figure 3(f)). Semiquantitative analysis confirmed the significant inhibition of inflammation by EA at the acupoints (*P* < .01 as compared with the FCA group; Figure 4), suggesting a specific role for EA in the control of pathological inflammatory responses.

### 3.3. Upregulation of the VIP mRNA Expression in the Synovial Tissue

**Figure 5:**
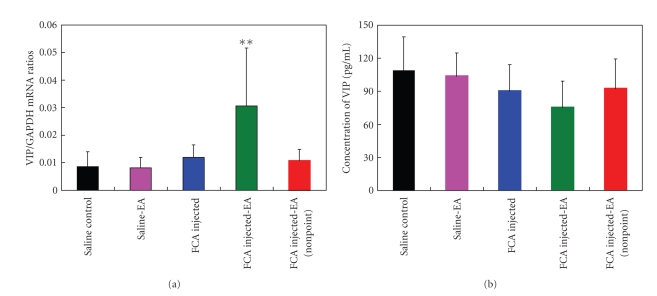
Examination of the serum and synovial VIP contents. (a) qPCR analysis of the expression of *VIP* mRNA in the synovial tissue. (b) No statistical difference in the serum VIP concentrations are found among the indicated groups. **P < .01, compared with the FCA group. Data are means ± SD (*n* = 15).

The results of qPCR analysis demonstrated a 2.6-fold higher amount of *VIP* mRNA in the synovial tissue in the FCA-EA group than in the FCA group (*P* < .01; Figure 5(a)). By contrast, the serum VIP protein concentration was slightly decreased following the EA treatment, but there was no statistical difference between the FCA and FCA-EA groups (Figure 5(b)). Additionally, no significant difference in the synovial *VIP *mRNA and serum VIP protein levels was detected among the saline, FCA, saline-EA, and FCA-EA (nonacupoint) groups.

### 3.4. Enhanced Immunostaining for VIP in the Synovial Tissue

**Figure 6:**
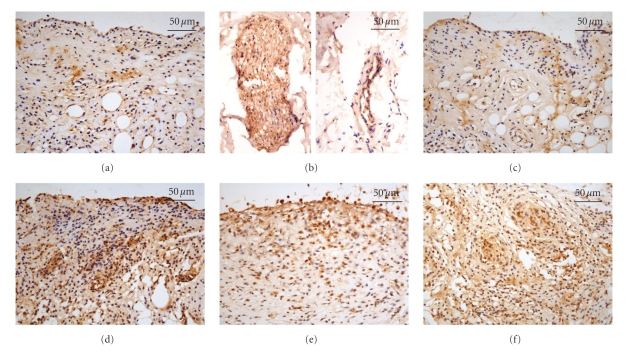
Immunostaining for VIP in the synovial tissue. Histological analysis of the synovial tissue from the saline group shows weak staining for VIP in synovial cells (a) and moderate staining for VIP in endothelium, smooth muscle, and nerve fibers (b). (c) Synovial cells from the saline-EA group display weak immunoreaction for VIP. (d) Moderate to strong immunostaining for VIP is seen in the synovial tissue from the FCA-injected animals. An enhancement of the VIP immunoreactivity in the synovial tissue is observed in the FCA-EA group (e) but not in the FCA-EA (nonacupoint) group (f). Original magnification, 400x.

**Figure 7:**
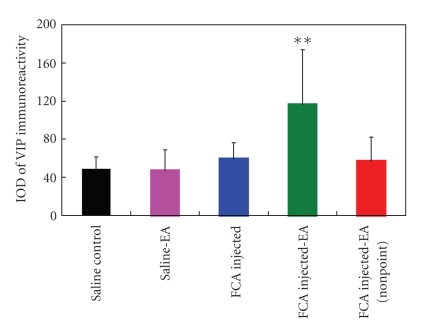
The integral optical density (IOD) of VIP immunoreactivity in the synovial tissue. **P < .01, compared with the FCA group. Data are means ± SD (*n* = 15).

Histological analysis of the synovial tissue from the saline group showed weak staining for VIP in synovial cells (Figure 6(a)) and moderate staining for VIP in endothelium, smooth muscle, and nerve fibers (Figure 6(b)). Likewise, the animals in the saline-EA group also showed weak immunoreaction for VIP in synovial cells (Figure 6(c)). Moderate to strong immunostaining for VIP was seen in the synovial tissue from the FCA-injected animals (Figure 6(d)). Most interestingly, the EA treatment resulted in a profound enhancement of the VIP immunoreactivity in the synovial tissue (Figure 6(e)). However, the electrical stimulation at nonacupoint had little influence on the VIP expression in the synovial tissue (Figure 6(f)). The results of the computer image analysis further demonstrated the EA treatment following FCA significantly increased the VIP expression in the synovial tissue in comparison with the FCA alone (116.46 ± 57.88 versus 59.27 ± 16.86, *P* < .01; Figure 7). No significant difference was detected among the saline, FCA, saline-EA, and FCA-EA (nonacupoint) groups.

### 3.5. The Relationship between the EA-Elicited Anti-Inflammatory Effects and the VIP Levels

**Figure 8:**
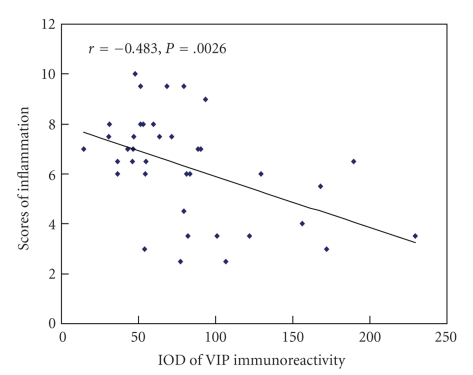
Relationship between the VIP immunostaining intensity and the scores of inflammation in the synovial tissue in rats with adjuvant-induced arthritis. *n* = 15.

To determine whether there is a positive or negative correlation between VIP expression and the scores of inflammation, Spearman bivariate correlation analysis was conducted. As shown in Figure 8, the VIP-immunostaining intensity was significantly negatively correlated with the scores of inflammation in the synovial tissue (*r* = −0.483, *P* = .0026), suggesting that the anti-inflammatory effects of EA at acupoint may be mediated through the up-regulation of VIP expression.

## 4. Discussion

In the present study, we established an FCA-induced arthritis model in rats and evaluated the effects of EA stimulation on the experimental arthritis. Our data show that the EA at acupoints (ST36, GB39 and BL23) remarkably reduced the body weight loss and alleviated the inflammation and paw swelling induced by FCA injection. In contrast, the EA stimulation at the above acupoints did not elicit any significant effects in saline-treated animals, suggesting a specific role for EA in the control of pathological inflammatory responses. Accompanying with the anti-inflammatory action, EA treatment resulted in an enhancement of VIP expression in the synovial tissue. Moreover, the VIP-immunostaining intensity was negatively correlated with the inflammatory scores in the synovial tissue. These findings collectively suggest a potentially important role for VIP in mediating the antiarthritic effect of EA.

As a traditional Chinese medical technique, acupuncture has become very popular worldwide in the treatment of various illnesses [1]. Several lines of evidence have demonstrated that it is effective in treating pain and dysfunction in patients with osteoarthritis of the knee [34, 35]. In a rat model of collagen-induced arthritis, bee venom acupuncture has been reported to be able to relieve inflammatory pain [36]. Our present data consistently indicate that EA at acupoints can control the FCA-evoked experimental arthritis. The EA effects seem to be acupoint specific, since the electrical stimulation at nonacupoint did not produce any beneficial outcome. This hypothesis is supported by a previous study [37], where immunomodulatory effects are exerted by the EA stimulation at acupoint ST36 but not at a nonacupoint (tail).

**Figure 9:**
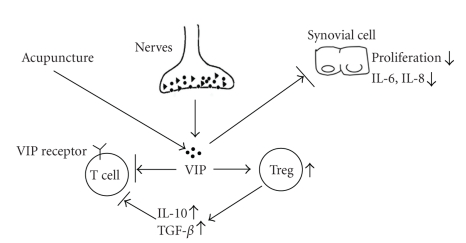
A hypothetical diagram illustrating the EA action mediated by VIP in adjuvant-induced arthritis. EA stimulation at specific acupoints promotes the expression and release of VIP, which in turn suppresses the production of proinflammatory cytokines and chemokines (e.g., MCP-1, IL-6, and IL-8) by synovial cells and/or induces regulatory T cells (Treg) expansion, ultimately leading to the inhibition of lymphocyte proliferation, activation, and infiltration and the alleviation of inflammatory responses.

The nervous system has been reported to be involved in the regulation of the pathogenesis of RA, through the production of several neuropeptides such as somatostatin, VIP, and calcitonin gene-related peptide [38]. VIP has been proved as a potent anti-inflammation molecule in several models of inflammatory disease [39]. Our data demonstrated that VIP likely act as an important mediator of the anti-inflammatory role of EA, which was supported by the findings that the EA treatment could markedly enhanced the expression of VIP in the synovial tissue, and moreover, the synovial VIP levels were negatively correlated with the inflammation scores. The migration of leukocytes into the synovial tissue is one of the hallmarks of chronic inflammatory rheumatic diseases such as RA. The involvement of VIP in the anti-inflammatory action of EA is likely through the inhibition of lymphocyte infiltration, since this neuropeptide is able to modulate the production of proinflammatory cytokines and chemokines by synovial cells in RA [38, 40]. Another alternative mechanism responsible for the VIP function is the modulation of autoreactive T cell activation/expansion through inducing a Th2-type response and expanding CD4^+^CD25^+^ regulatory T cells [21]. A hypothetical diagram of the anti-inflammatory action of EA mediated by VIP in RA is shown in Figure 9.

In conclusion, our results indicated, for the first time, that the EA treatment at Zusanli (ST 36), Xuanzhong (GB39), and Shenshu (BL23) could decrease the body weight loss and suppress the paw edema and inflammatory reaction in rats with Freund's adjuvant-induced arthritis, at least partially, via upregulating the VIP expression. These results might provide an alternative therapy for RA. Further studies will be necessary to determine the extent to which the present findings are applicable to RA patients.
